# Case report: Papillary mesothelioma of the peritoneum with foamy cell lining

**DOI:** 10.1186/1746-1596-8-162

**Published:** 2013-09-25

**Authors:** Simona Stolnicu, Enoe Quiñonez, Monica Boros, Claudiu Molnar, Isabel Dulcey, Francisco F Nogales

**Affiliations:** 1 Department of Pathology, University of Medicine and Pharmacy, Str. Gh. Marinescu nr 38, Targu Mures, Romania; 2 San Cecilio University Hospital, Avenida Madrid 11, Granada, Spain; 3 Department of Gynecology, University of Medicine and Pharmacy, Str. Gh. Marinescu nr 38, Targu Mures, Romania

**Keywords:** Mesothelioma, Papillary, Foamy cell change, Immunohistochemistry

## Abstract

A 34-year-old female, with a history of continued asbestos exposure, presented with a papillary peritoneal mesothelioma with a diffuse, prominent clear foamy cell change, with microvacuolation in its papillary lining, that expressed cytokeratins 7, 5/6 and calretinin as well as nuclear WT-1 and apical membrane staining for thrombomodulin, podoplanin D2-40 and HBME-1. In contrast, lining cells were CD68 negative. Foamy cell change has been reported in isolated cases as solid cords but not as a diffuse change in the mesothelial papillary lining. This phenomenon prompts differential diagnoses with abdominal and renal papillary clear cell tumours, which were discarded after a characteristic mesothelial immunophenotype was demonstrated.

**Virtual slides:**

The virtual slide(s) for this article can be found here: http://www.diagnosticpathology.diagnomx.eu/vs/4679576081031834.

## Background

Malignant mesothelioma of the peritoneal cavity accounts for only 10-20% of all mesotheliomas and usually involves elderly or middle age males, often with a previous history of asbestos exposure [1]. The occurrence of this tumour in young women is rare [2] and related with lesser frequency to asbestos exposure [3,4]. Histologically, most cases correspond to the epithelial type with an admixture of tubular, papillary and solid growth patterns, although biphasic or sarcomatous types can also occur.

Foamy tumour cells forming solid sheets, have been described in three cases of malignant mesothelioma of the peritoneum [1,5], two in the pleura and one of the tunica vaginalis testis [6-8]. However, foamy cell change has not been described in the papillary lining. We report here such a case in a patient exposed to asbestos, where a diffuse, prominent clear foamy cell change prompted various differential diagnoses with abdominal and renal papillary clear cell tumours, which were discarded after a characteristic mesothelial immunophenotype was demonstrated.

## Case presentation

A 34-year-old non-smoking female, with a history of 30 years of continued exposure to roofing and wall asbestos fibrocement plates (composed by 90% and 10% asbestos fibers), presented to the Gynaecology Department, Oradea District Hospital, Romania, with abdominal distension and pain. Neither the patient nor her close family member is an active or passive smoker. A computerized tomography scan showed abundant ascites and multiple intra-abdominal nodules ranging from 2-10 cm. An elevated serum CA125 was present. Abdominal surgery revealed a 10 cm lobulated, friable omental growth and other multiple smaller nodules in the parietal peritoneum. The right ovary was unremarkable, while the left one showed 2-5 mm nodules on its surface. Omentectomy, partial left ovary resection and debulking of the largest nodule in the peritoneum were performed. The patient unfortunately declined any further oncologic treatment and she is alive with tumour 3 months after the initial diagnosis.

## Materials and methods

All the 10 tumour tissue samples were formalin fixed, paraffin-embedded and stained with H&E. Standard immunohistochemical protocols performed for the following antibodies are shown in Table 1.

**Table 1:** Antibodies used in this study

**Antibody**	**Dilution**	**Clone**	**Vendor**
Cytokeratin 7	Prediluted,	OV-TL12/30	Masterdiagnostica, Granada, Spain.
Cytokeratins 5/6	Prediluted	D5/16B4	Masterdiagnostica, Granada, Spain.
WT1	Prediluted	6F-H2	DakoCytomation, Denmark
Calretinin	Prediluted	DAK-Calret1	DakoCytomation, Denmark
Thrombomodulin	Prediluted,	1009	DakoCytomation, Denmark
Podoplanin	Prediluted,	D2-40	DakoCytomation, Denmark
Anti-mesothelioma	Prediluted	HBME-1	DakoCytomation, Denmark
CD68	Prediluted	PG-M1	DakoCytomation, Denmark

## Results

**Figure 1:**
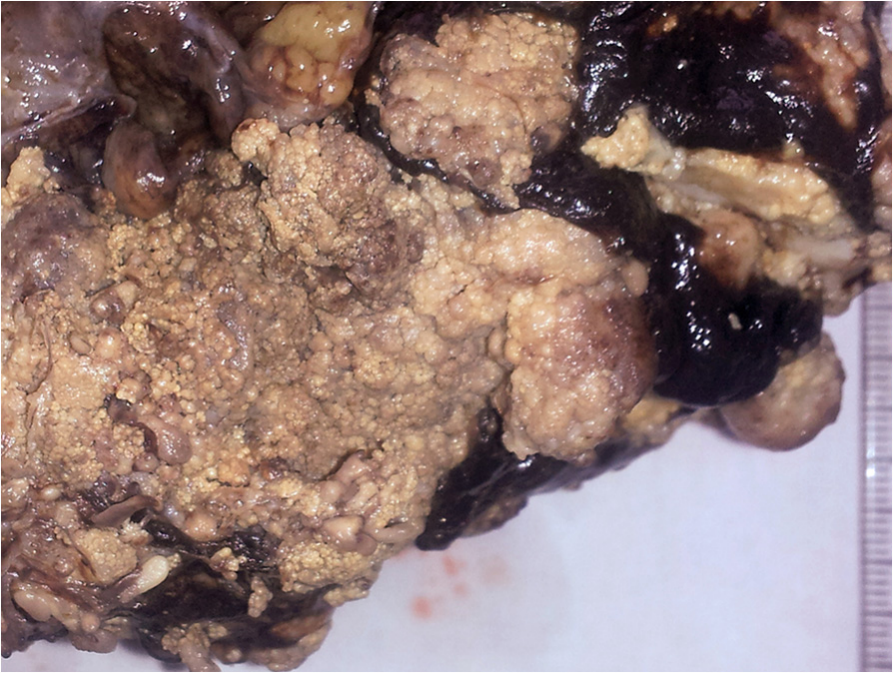
Omental mass with a grainy yellow texture (codfish roe-like).

**Figure 2:**
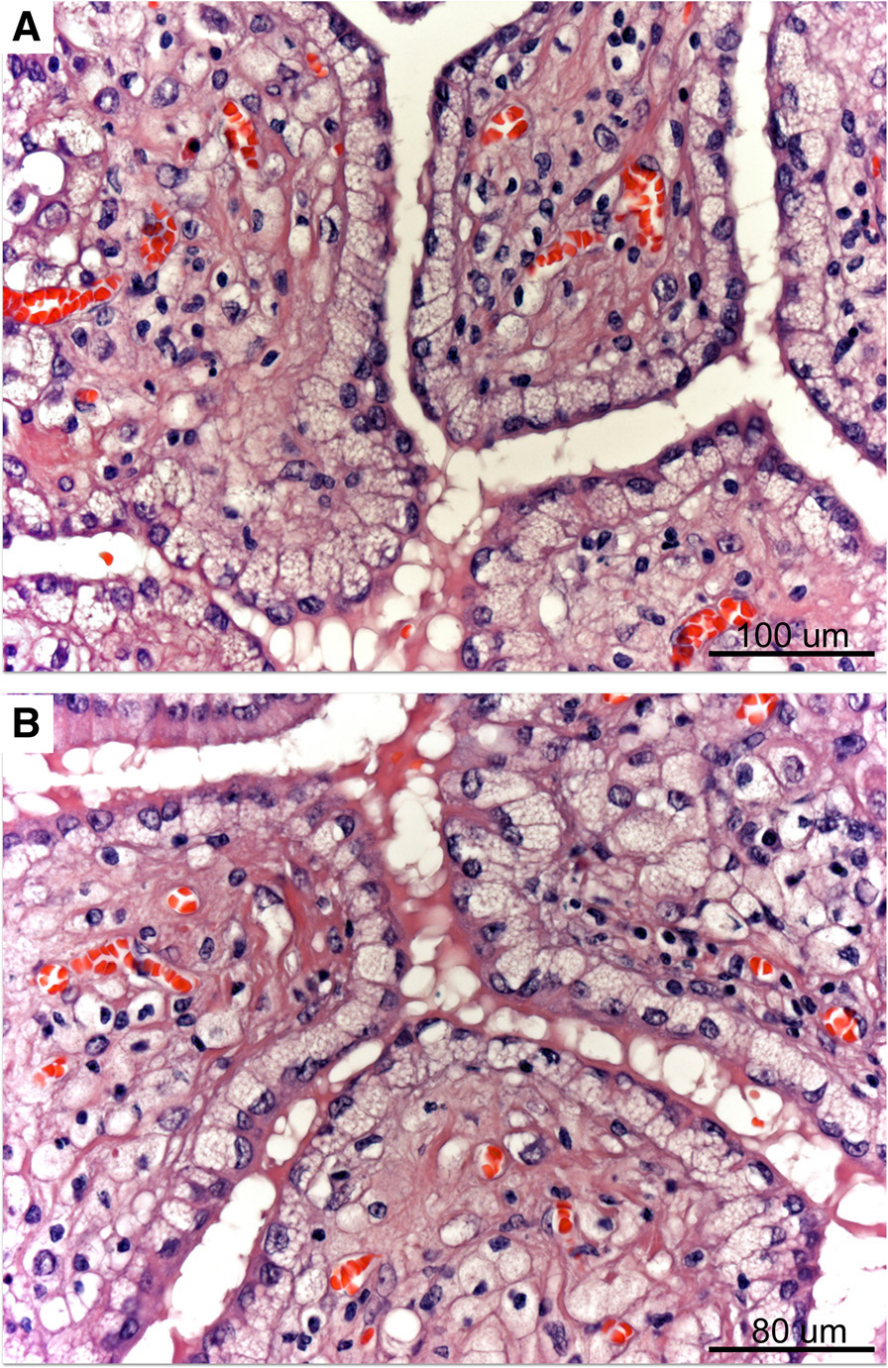
**Papillary mesothelioma with a clear, foamy cell lining (A**&**B).** Note basal microvacuolation displacing nuclei towards the surface. Cords of clear cells are present in the core.

On cut section, the larger mass had a grainy yellow appearance, reminiscent of codfish-roe (Figure 1). Histologically, all samples displayed a similar appearance showing a complex branching papillary architecture lined by tall, cylindrical atypical cells with a basal clear basophilic, foamy cytoplasm stippled by abundant microvacuoles that displaced the nuclei towards the periphery (Figure 2a-b). Vacuolated cells were seen as isolated cords in the papillary cores. Plump eosinophilic or hobnail-type cells were also seen in smaller amounts.

**Figure 3:**
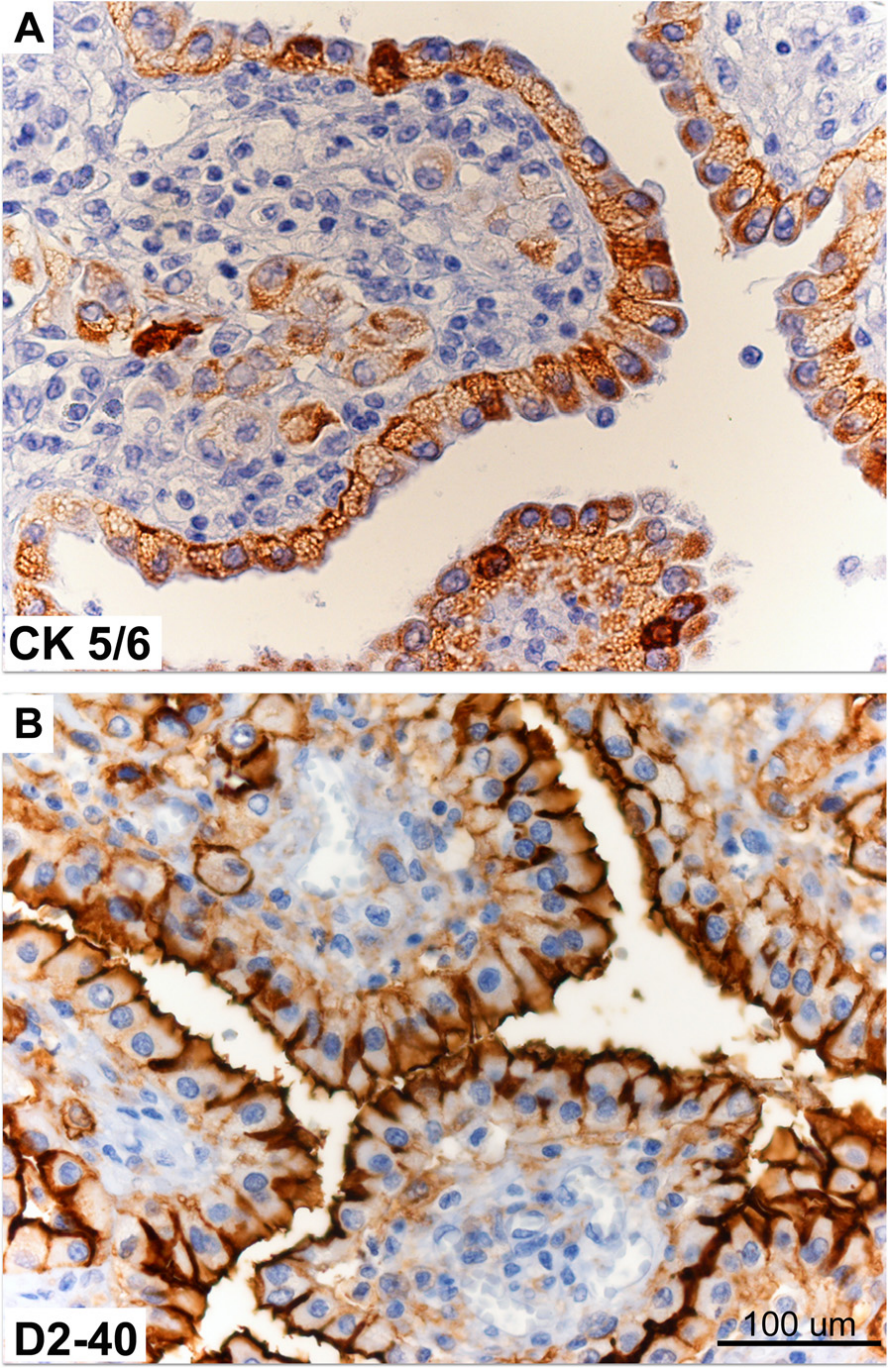
Immunohistochemical expression of CK5/6 delineates diffusely epithelial cytoplasmic microvacuoles (A), while D2-40 (B) is only apically expressed.

Immunohistochemically, all tumour cells (both foamy and eosinophilic cells) expressed diffusely cytoplasmic cytokeratin 7, cytokeratins 5/6 (Figure 3a) and calretinin. Furthermore, additional characteristic mesothelioma markers such as WT-1, showed nuclear expression, while thrombomodulin, podoplanin D2-40 (Figure 3b) and HBME-1 revealed strong apical membrane staining. In contrast, CD68 was negative in all tumour cells being only positive in clusters of histiocytes found in the papillary stroma. The final diagnosis was of a malignant mesothelioma of the peritoneum of foamy cell subtype involving the omentum and left ovarian surface.

## Discussion

This unusual histological variant of mesothelioma exhibits a diffuse cytoplasmic microvacuolar foamy change. Seven cases, including the present one, have been published in the literature [1,5-9]. All cases (Table 2) correspond to the usual locations of mesothelioma: abdomen (three cases), pleura (two cases) and testis (one case), with a predominance of abdominal locations in females. Three cases including the present one were associated with asbestos exposure and one had a previous history of trauma. In this case, a continuous exposure of more than 30 years to asbestos fibers present in the roof plates of the house was found. This is in agreement with previous published studies that show a latency period after asbestos exposure and eventual mesothelioma development ranging from 4–40 years, although cases with a shorter period have also been reported [10,11]. Clinically, their behaviour was similar to the usual types of mesothelioma. Histologically, all cases except ours, illustrated the presence of these foamy cells only in solid sheets of stromal tumour. The present case, however, was different since it revealed a predominant, diffuse foamy cell change throughout the epithelial lining of the papillae and isolated cords of neoplastic foam cells in the papillary cores.

**Table 2:** Review of published cases of foamy cell mesothelioma

**Case**	**Age, gender, site**	**Clinical data**	**Microscopy**	**Treatment**
Cavazza [12]	56 F	Asbestos exposure	Papillary, 40% foam cells in solid areas	Died 6 mo.
	Pleura			
Mikuz [4]	18 M	Traumatic hydrocele	Papillary. Foam cells in solid areas	Surgery
	Testis			Alive and well 17 mo.
Ordoñez [2]	73 M	Asbestos exposure	Papillary. Foam cells in solid areas & carcinoid tumor	Chemotherapy and surgery
	Pleura			Died 6 mo.
Baker (series) [13]	47 F	-	Papillary. Foam cells in solid areas	-
1 case/75	unknown			
Komorowski [14]	45 F	-	Papillary. Foam cells in solid areas	Surgery and radiation
	Abdominal			Died 1 yr.
Kitazawa [10]	31 F	-	Papillary. Solid stromal foamy cell	Surgery and radiation.
	Abdominal			Alive and well 5 yr.
Present case	34 F	Asbestos exposure	Papillary. Foam cells in papillae and cores	Surgery 3 mo.
	Abdominal			

A curious macroscopic feature of this tumour variant, which was also present in this case, is represented by its granular yellow texture, which has been compared to codfish roe [5], possibly related to its papillary arrangement and oxidative change of lipids.

Electronmicroscopic studies have shown [5,6] that foamy cells harbour degenerative phaenomena [5] and are different from histiocytes, a fact confirmed by the absence of CD68 expression in the present case. A similar change has been described in desquamated mesothelial cells of pleuroperitoneal effusions [12]. Analogous clear, foamy tumour cells may occur in rare tumours of other locations such as kidney [14,15], prostate [16,17] and pancreas [13] that should be considered in the differential diagnosis of this tumour. Only rarely, ovarian clear cell carcinomas or mucinous tumours may exhibit isolated foamy epithelial cells.

Usually in a malignant mesothelioma of peritoneum, the tumour cells retain a resemblance to mesothelium. Sometimes the tumour cells may resemble decidual, hobnail-type and rhabdoid cells, with clear cells or foamy cells being unusual. In Baker et al. [1] series of 75 cases of malignant mesothelioma of peritoneum, a single 47 year old patient showed the presence of foamy tumour cells.

Differential diagnosis, however, is facilitated by the identification of a characteristic mesothelioma immunophenotype [14,18], expressing calretinin, CK 5/6, WT1, thrombomodulin, mesothelin, HBME-1 and podoplanin D240, features absent in cytologically similar neoplasms (in both females and males) which exhibit their characteristic markers. Correspondingly, clear-cell papillary renal cell carcinomas [14], are positive for CD10 and negative for cytokeratin 7, prostatic carcinomas with abundant xanthomatous cytoplasm [16] show a strong prostate specific antigen positivity and finally, foamy gland patterns of pancreatic ductal adenocarcinoma [13] are diffusely positive for carcinoembryonic antigen, cytokeratin 8 and MUC1.

## Conclusions

Foamy cell change has been reported in isolated cases of mesothelioma as solid cords but not as a diffuse change involving the mesothelial papillary lining. This phenomenon would prompt differential diagnoses with abdominal tumours such as clear cell carcinoma of the ovary and renal papillary clear cell tumours, which can display similar lining of their papillae. However, the characteristic mesothelial immunophenotype helps in establishing a differential diagnosis.

## Consent

Written informed consent was obtained from the patient for publication of this Case Report and any accompanying images. A copy of the written consent is available for review by the Editor-in-Chief of this journal.

## Competing interest

The authors declare that they have no conflict of interest.

## Authors’ contribution

SS, MB, CM performed the original diagnosis and clinicopathological analysis. EQ and ID performed the immunohistochemistry and composed the illustration. SS and FFN drafted and corrected the manuscript. All authors read and approved the final manuscript.
